# A Novel Variant in the DIAPH1 Gene Causing Macrothrombocytopenia and Non-syndromic Hearing Loss in a Pediatric Saudi Girl

**DOI:** 10.7759/cureus.61044

**Published:** 2024-05-25

**Authors:** Badriah G Alasmari, Mohammed Alpakra, Sara S Hassanien, Abdelhakam A Elmugadam, Lina Elzubair, Enaam A Suliman, Somayah A Alghubishi

**Affiliations:** 1 Pediatric Hematology and Oncology, Armed Forces Hospitals Southern Region, Khamis Mushayt, SAU; 2 Pediatric Hematology and Oncology, Armed Forces Hospitals Southern Region, Khamis Mushait, SAU; 3 Hematopathology, Armed Forces Hospitals Southern Region, Khamis Mushayt, SAU; 4 Pediatrics, Armed Forces Hospitals Southern Region, Khamis Mushayt, SAU

**Keywords:** novel gene, platelets, hearing loss, non-syndromic, macrothrombocytopenia

## Abstract

Macrothrompocytopenia (MTP) is a rare group of hereditary disorders that lead to impaired hemostasis. Macrothrompocytopenia mostly results from genetic mutations in genes implicated in megakaryocyte differentiation and function. Diaphanous-related formin 1 (DIAPH1) is a protein-coding gene. Dominant gain-of-function DIAPH1 variants cause macrothrombocytopenia and sensorineural deafness (autosomal dominant non-syndromic hearing loss 1 (DFNA1)), while homozygous loss of DIAPH1 results in seizures, cortical blindness, and microcephaly syndrome (SCBMS). This rare genetic disease is characterized by progressive and severe hearing loss with onset in the first decade of life, is associated with mild thrombocytopenia, and has no significant bleeding tendency. This case report presents the clinical findings of a 14-year-old Saudi pediatric girl. We investigated the potential association of DIAPH1 as a novel candidate gene linked to dominant MTP and autosomal dominant non-syndromic hearing loss (ADNSHL), which was evaluated through audiometry. Notably, a novel variant, c.3633_3636del, was identified in the DIAPH1 gene. To date, only a small number of mutations in this gene have been reported as the cause of MTP and ADNSHL.

## Introduction

Platelets are produced when mature megakaryocytes (MKs) release proplatelets, which are cytoplasmic extensions, into the bone marrow sinusoids. These sinusoids are responsible for the ultimate size and morphology of platelets [1]. Several human hematopoietic disorders are characterized by altered regulation of platelet formation, including macrothrombocytopenia (MTP), which causes abnormal bleeding and involves a reduction in the number of circulating platelets [1]. This phenotype results from MKs' altered regulation of platelet formation [1]. Pathogenic variants in genes that modulate MK, including the diaphanous-related formin 1 (DIAPH1) gene, have been linked to MTP [1].

The DIAPH1 gene encoded a protein that was truncated. Diaphanous-related formin 1, a cytoskeletal regulator and Rho effector that regulates megakaryocytopoiesis is encoded by this gene. A diaphanous autoregulatory domain located at the carboxy terminus regulates DIAPH1; its activity is inhibited by a diaphanous inhibitory domain (DID) connected to DIAPH1 at the amino terminus [2]. In the absence of the DAD domain, the truncated protein is constitutively active, leading to an accumulation of filamentous actin and the formation of stable microtubules [2].

The DIAPH1 gene is additionally expressed in inner ear hair cells. Thus, autosomal dominant MTP and hearing loss result from this gain of function mutation, as opposed to autosomal recessive microcephaly brought about by the biallelic truncating DIAPH1 mutation. [3] 1997 marked the identification of DIAPH1 as the initial causative gene of autosomal dominant non-syndromic hearing loss (ADNSHL) in a sizable Costa Rican relative who presented with profound, bilateral deafness encompassing all frequencies by the time they were 30 years old, whereas low-frequency deafness commenced around the age of ten. Despite the likely progression of hearing loss and MTP, no evidence of clinically significant hemorrhage was detected [4].

## Case presentation

**Figure 1:**
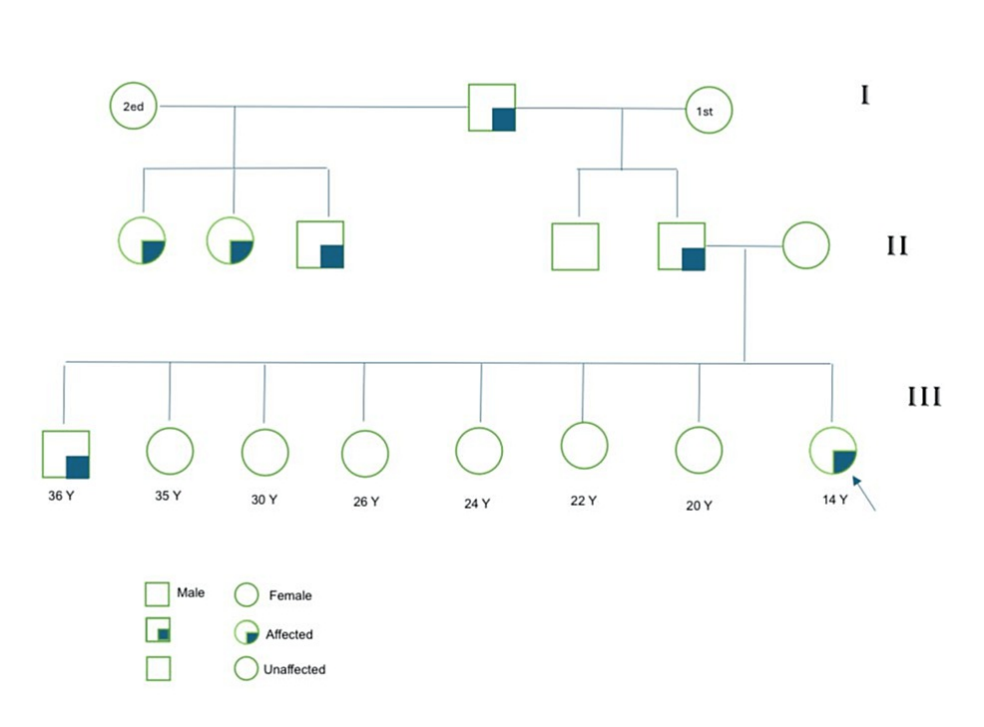
The pedigree of the family shows no family affected, apart from one aunt with hearing loss. y: years

This case study presents the case of a 14-year-old pediatric Saudi girl referred from the otolaryngology clinic due to low platelet counts discovered during routine preoperative screening for cochlear implantation. The patient had a history of hearing problems since age three, which were managed through regular follow-up. By age 10, she had developed bilateral hearing loss, requiring cochlear implantation in the right ear with surgery planned for the left ear. Notably, she exhibited normal growth and development despite being born to consanguineous parents. Her family history revealed hearing problems in three members of her paternal family, specifically her three paternal aunts. Conversely, there were no cases of hearing issues or low platelet counts on the father's side of the family. Furthermore, no history of bleeding, bruising, or hearing problems was observed in her three sisters. Family members' blood counts and smears were normal, except for giant platelets in three sisters (Figure 1).

Investigations revealed a low platelet count ranging from 68,000-111,000/uL, accompanied by normal hemoglobin levels (13.6 gm/dL) and white blood cell counts (5100 cells/μL) (Table 1). Liver function tests showed unremarkable results, and no protein was detected in the urine. Furthermore, the erythropoietin levels were within normal limits.

**Table 1:** Complete blood count (CBC) results and reference ranges for the case *H: high; L: Low WBC: white blood cell count; RBC: red blood cell count; HCT: hematocrit; MCV: mean corpuscular volume; MCH: mean corpuscular hemoglobin; MCHC: mean corpuscular hemoglobin concentration; RDW: red cell distribution width; PLT: platelet count; MPV: mean platelet volume; NEUT%: neutrophil percentage; LYMPH%: lymphocyte percentage; MONO%: monocyte percentage; EOS%: eosinophil percentage; BASO%: basophil percentage; LUC%: immature granulocyte percentage; NEUT ABS: absolute neutrophil count; LYMPH ABS: absolute lymphocyte count; MONO ABS: absolute monocyte count; EOS ABS: absolute eosinophil count; BASO ABS: absolute basophil count (BaSO ABS); LUC ABS: absolute immature granulocyte count

Description	Result	Units	Reference range
WBC	5.10	10^9^/L	4.5 – 13.5
RBC	5.66 ^*H^	10^12^/L	4.1 – 5.3
Hemoglobin	13.3	g/dl	10.9 – 15
HCT	44.0	[%]	34 – 44
MCV	77.7	fl	76 – 96
MCH	25.3	pg	23 – 30
MCHC	32.5	g/dl	32 – 36
RDW	15.9 ^*H^	[%]	11 – 14
PLT	68	10^^9^/L	N/A
MPV	12.3	fl	9.4 – 12.3
NEUT%	28.80	%	N/A
LYMPH%	58.50	%	N/A
MONO%	6.90	%	N/A
EOS%	1.40	%	N/A
BASO%	0.90	%	N/A
LUC%	3.60	%	N/A
NEUT ABS	1.57 ^*L^	10^3^/mL	1.8 – 8
LYMPH ABS	3.19	10^3^/mL	1.5 - 6.5
MONO ABS	0.37 ^*L^	10^3^/mL	0.4 - 2
EOS ABS	0.07 ^*L^	10^3^/mL	0.2 – 1.9
BASO ABS	0.04	10^3^/mL	N/A
LUC ABS	0.19	10^3^/mL	N/A

**Figure 2:**
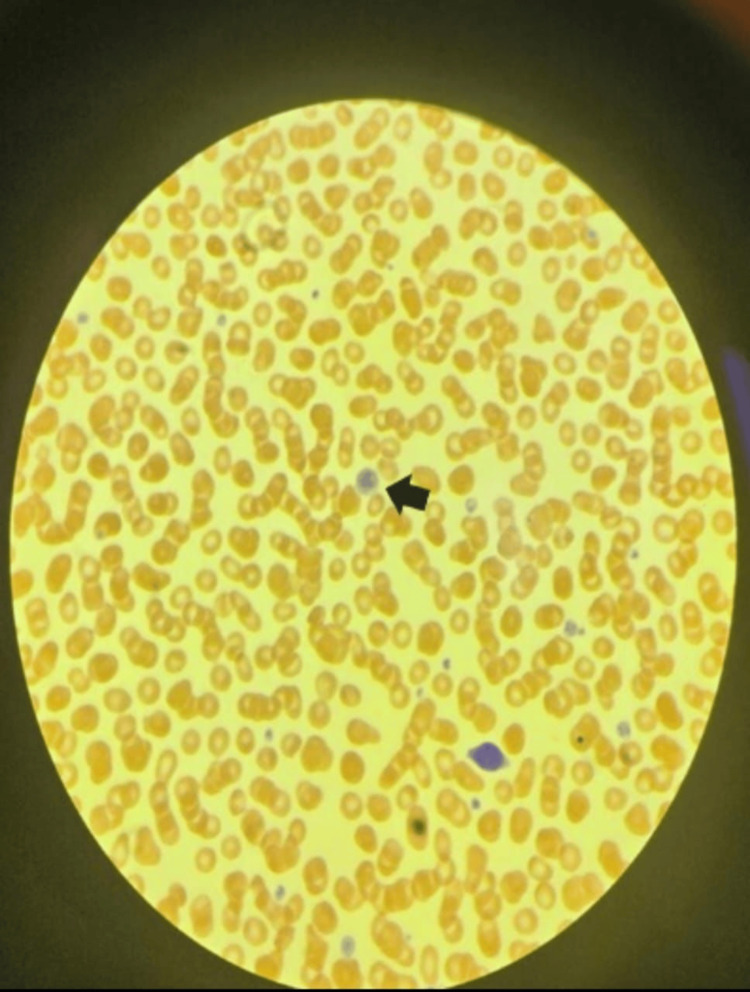
Peripheral smear analysis Representative peripheral blood smear (PBS x100) stained with Wright-Giemsa. Findings include hypochromic macrocytic red blood cells (RBC) +2, anisopoikilocytosis +1, mild neutropenia, absence of blasts, and moderate thrombocytopenia characterized by a mixture of normal-sized platelets with occasional giant platelets. The arrow indicates a manual platelet count of 90 x 109/L, with no presence of platelet clumps.

Peripheral smear analysis showed moderate thrombocytopenia with a mixture of normal and large platelets (Figure 2).

Whole exome sequencing identified a novel heterozygous variant c.3633_3636del in the DIAPH1 gene with a resultant amino acid change of p. (Phe1212Aspfs*21) (Table 2). The zygosity parameter indicated a homozygous state (WA). Notably, this variant is classified as having uncertain significance according to recommendations by CENTOGENE and the American College of Medical Genetics and Genomics (ACMG)/American Association of Molecular Pathology (AMP) ClinGen Sequence Variant Interpretation Working Group (SVI) general recommendations. It appears to be a novel variant not previously reported in the literature. Our patient also tested negative for MYH9 gene. The patient is now under regular follow-up with pediatric hematology, otolaryngology, and speech therapy, showing positive responses.

**Table 2:** Whole exome sequencing study MIM: Mendelian Inheritance in Man; AD: autosomal dominant; Het: heterozygous

Gene	Variant	Zygosity	MIM
DIAPH1	NM_005219.5:c.3633_3636del	Het	AD

## Discussion

Macrothrombocytopenia disorders are a group of rare diseases that affect a small number of individuals. The estimated incidence of these disorders is 270 cases per million live births [5]. At first, the classification of MTP based on genetics was done by studying specific genes in groups of patients who had similar clinical or laboratory characteristics. They used Sanger sequencing to determine their genotype [5]. As of 2004, there were only nine genes identified with etiological mutations for MTP disorders [6]. However, the emergence of high-throughput sequencing technology has made it easier to conduct extensive testing using diagnostic gene panels for MTP [5]. In addition, extensive research studies using whole genome sequencing and whole exome sequencing have greatly increased our understanding of MTP genes and improved our knowledge of the harmful variations within these genes [7].

One more recently discovered MTP gene is DIAPH1. The DIAPH1 gene encodes a member of the formin protein family, responsible for regulating the GTPase-dependent assembly of actin and microtubule dynamics during cytoskeletal remodeling [1]. Variants associated with DIAPH1-related macrothrombocytopenia result in the constant activation of the DIAPH1 protein, leading to disruption of cytoskeletal and microtubule function, thereby impairing proplatelet formation [1]. Patients with rare causative variants in DIAPH1 have moderate macrothrombocytopenia without bleeding complications, but often mild neutropenia and severe sensorineural deafness can also be observed [8]. Our pediatric patient exhibited moderate thrombocytopenia without associated bleeding complications, along with hearing loss necessitating cochlear transplantation.

The prevalence of hearing loss ranges from 2.8 per 1,000 in school-aged children to 3.5 per 1,000 in pediatrics [9]. Genetic causes account for 50%-60% of hearing loss in children, and a genetic cause should always be investigated for any individual with a hearing issue, even if additional environmental risk factors exist [9]. Hereditary hearing loss can be syndromic (i.e., occur with other symptoms) or non-syndromic. More than 70% of genetic hearing loss cases are classified as non-syndromic, showcasing considerable genetic and clinical diversity [10]. While the majority of non-syndromic hearing loss cases are inherited in an autosomal recessive manner (75%-80%), X-linked (2%-5%), mitochondrial mutations (1%), and autosomal dominant (20%), they can also contribute to hearing loss [11]. Our patient also suffered from non-syndromic hearing loss.

Hearing loss associated with DIAPH1 typically begins in the low-frequency region. It progresses throughout the entire frequency range, starting in the first 10 years of life and advancing rapidly to a profound degree by the fourth decade [12,13]. While non-syndromic hearing loss is usually not accompanied by other clinical appearances, certain autosomal dominant loci, such as autosomal dominant non-syndromic hearing loss 1 (DFNA1), may present with additional signs like thrombocytopenia [14,15]. Some individuals with DFNA1 may exhibit mild thrombocytopenia and enlarged platelets, although significant bleeding tendencies are uncommon [16,17]. While most individuals diagnosed with ADNSHL have a parent with hearing impairment, it's important to note that a negative family history may result from late-onset hearing loss in a parent, the presence of a de novo variant, or decreased pathogenic variant penetrance [18,19]. In our case, hearing loss was observed in two of the patient's maternal aunts. Additionally, she and her three sisters exhibited large platelets.

As one of the most effective treatments for hearing loss, cochlear implantation is dependent on the identification of the responsible genes that determine which region of the cochlea is affected; implantation can only be effective if the mutated lesion lies in afferent synapses between the auditory nerve and hair cells or directly in the hair cells themselves [20].

## Conclusions

Macrothrombocytopenia is often identified in asymptomatic individuals through routine laboratory examinations. In routine laboratory examinations, a peripheral blood smear must be conducted to detect abnormalities in platelets and neutrophilic counts. The abnormalities in platelets, especially the presence of giant thrombocytes, serve as an indicator for the hereditary form of this condition.

Additionally, it is imperative that early genetic mutation screening be conducted on all children who have been diagnosed with sensorineural hearing loss in order to facilitate the timely administration of appropriate treatments such as cochlear implants, hearing aids, and individualized rehabilitation programs. Usually, a large number of children with hearing loss go undiagnosed during their crucial phase of speech development. As a result, early detection of genetic hearing loss seems to be a critical concern in order to detect possible comorbidities and direct treatment strategies.
